# Genetics of sudden death

**Published:** 2010-11

**Authors:** Nitish Naik, Rakesh Yadav

**Affiliations:** *Department of Cardiology, All India Institute of Medical Sciences, New Delhi, India*

**Keywords:** Arrhythmias, coronary artery disease, genomics, Ion channels, sudden death

## Abstract

Recent advances in molecular biology have advanced our understanding of the genetic substrate predisposing to sudden death, especially in monogenic disorders. Numerous ion channels along with membrane structural proteins have been extensively investigated for their role in the genesis of serious ventricular tachyarrhythmias. The complex interplay of various biological pathways culminating in the more prevalent form of sudden death due to coronary artery disease however still remains to be unraveled. The concept of multi-factorial causation of arrhythmias where a second clinical or environmental factor is necessary for expression of an underlying genetic susceptibility to ventricular arrhythmias is a serious possibility. This article briefly outlines the current understanding about the role of genetics in sudden cardiac death.

## Introduction

Sudden death is estimated to account for nearly 450,000 deaths annually in the United States, which represents a prevalence of 0.1 per cent in the general population^1^. Ventricular tachyarrhythmias (either ventricular fibrillation or ventricular tachycardia) are responsible for disordered ventricular contraction resulting in sudden death in most of the cases. Coronary artery disease (CAD) is the major underlying substrate responsible for sudden death - in fact, majority of such events are the presenting manifestation of previously quiescent CAD^2^. Myocardial diseases such as dilated cardiomyopathy and hypertrophic cardiomyopathy along with primary electrical diseases of the heart such as long QT syndrome, Brugada syndrome, idiopathic ventricular fibrillation, *etc*. account for about 15–20 per cent of sudden deaths^2^. In patients with clinically manifest coronary artery disease, clinical parameters such as reduced ventricular function, history of heart failure, frequent ventricular ectopy, inducible ventricular tachycardia on electrophysiologic studies, and abnormal autonomic function tests such as heart rate variability and abnormal microvolt T wave alternans are markers for an increased probability of sudden death^3^. One of the strongest clinical predictors of occurrence of sudden death in these patients is a parental history of sudden death^4^, and genes could play a direct role at least in some causes of sudden death. Many studies have documented this genetic influence on occurrence of sudden death^4^–^10^. This article reviews the recent advances about the role of genetics in sudden death.

## Role of genes in arrhythmogenesis due to monogenic disorder

The most definitive role for genes in arrhythmogenesis came from the study of monogenic disorders that result in sudden death. Primary electrical diseases such as long QT syndrome, Brugada syndrome, short QT syndrome and catecholaminergic polymorphic ventricular tachycardia are inherited disorders with a strong genetic basis^5^. Together these disorders account for approximately 5 per cent of sudden deaths in the population. These disorders result from mutations in single genes that adversely affect cardiac ion channel function, structural proteins associated with cardiac ion channels, or proteins affecting calcium handling. These alterations predispose the individual to unstable ventricular arrhythmias leading to sudden death^2^. In some syndrome subtypes, the exact mechanism is still not known.

Long QT syndromes are rare autosomal dominant inherited syndromes affecting approximately 1 in about 2500 individuals, and are produced by more than 400 different mutations^5^,^6^. Ten different subtypes have been described along with two autosomal recessive syndromes (Table). The gene product in eight of these syndromes codes for an ion channel protein while it codes for adaptor proteins in the remaining two syndromes. Long QT1 syndrome is the commonest subtype followed by long QT2 and then long QT3. Long QT4 and 5 syndromes account for approximately 1 per cent of all cases. The remaining subtypes are comparatively rare. All these mutations result in an abnormal prolongation of ventricular action potential duration that leads to early after depolarizations and torsades. In the long QT 1 syndrome for instance, a mutation in the alpha subunit of potassium channel protein KvLQT1 leads to a reduction in the inward potassium current, I_ks_. In long QT2 syndrome, mutation in another protein, human ether-a-go-go related gene (HERG), leads to a reduction in another inward potassium current, I_kr_. Long QT3 syndrome results from an increase in sodium channel function due to mutation in a protein Nav1.5 coded by the gene SCN5A. Majority of these patients present in childhood although some patients may be diagnosed for the first time in adulthood. In others long QT syndrome may manifest after exposure to drugs such as anti-histamines, quinolones, macrolide antibiotics, *etc*. This is an example of a “second hit” where an environmental insult is needed for the genetic abnormality to manifest. Although long QT syndrome runs in families, there may be significant variations in clinical features among family members due to variable penetrance (differing frequency of manifestation of genetic traits by individuals). Priori *et al*^7^ reported that penetrance of the long QT phenotype may be as low as 25 per cent in families with sporadic cases of long QT syndrome. This has significant implications because many apparently healthy individuals who harbour a long QT genotype may not have QT prolongation manifested and yet may be prone to sudden death. In addition, such an individual has a 50 per cent probability of transmitting this genotype to his/her offspring. Molecular genetics would, therefore, be required to more effectively exclude silent long QT genotype in such apparently healthy individuals.

**Table:** Long QT and Jervelle Lange-Nielsen syndromes: Genetic defects and channel abnormalities

Syndrome	Gene	Function
Autosomal dominant		
LQT1	*KCNQ1*	*I_ks_* Decreased
LQT2	*KCNH2*	*I_Kr_* Decreased
LQT3	*SCN5A*	*I_Na_* Increased
LQT4	*ANK2*	*I_Na, K_* Decreased
LQT5	*KCNE1*	*I_ks_* Decreased
LQT6	*KCNE2*	*I_kr_* Decreased
LQT7	*KCNJ2*	*I_k1_* Decreased
LQT8	*CACNA1C*	*I_Ca, L_* Increased
LQT9	*CAV3*	*I_Na_* Increased
LQT10	*SCN4B*	*I_Na_* Increased
Autosomal recessive		
JLN1	*KCNQ1*	*I_ks_* Decreased
JLN2	*KCNE1*	*I_ks_* Decreased

Cardiac sodium (*I_Na_*), Potassium (*I_ks_, I_Kr_, I_k1_*) and Calcium currents (*I_Ca, L_*)

Brugada syndrome is another rare syndrome that results from disease of ion channel fluxes in the ventricular myocardium. It has been associated with a loss of function of cardiac sodium channels coded by the gene *SCN5A*. Recently, another mutation in a gene product that codes for glycerol-6-phosphate dehydrogenase like protein has been associated with loss of function of cardiac sodium channels. These mutations result in current transients from ventricular epicardium to endocardium which culminate in ventricular tachyarrhythmias.

Cardiomyopathies such as hypertrophic cardiomyopathy, dilated cardiomyopathy and arrhythmogenic right ventricular dysplasia account for approximately 10–15 per cent of sudden deaths^2^. Genetic variations that affect cardiac structural sarcomeric proteins such as beta myosin heavy chain and cardiac troponins in patients with hypertrophic cardiomyopathy have been associated with increased risk of sudden death. Similar genetic variations in structural proteins in dilated cardiomyopathy and arrhythmogenic right ventricular dysplasia also predispose individuals to an elevated risk of sudden death due to reentrant ventricular tachyarrhyhmias^2^.

### Role of genes in coronary heart disease

While the role of genes in these rare monogenic disorders is more clearly established, the contribution of a person’s genetic make-up in determining the risk of sudden death due to the more common form of sudden death secondary to coronary artery disease is less clear. Sudden arrhythmic death in patients with coronary artery disease is a result of ventricular tachycardia/fibrillation secondary to acute ischaemia or due to myocardial scars^8^. Thus a multitude of genetic factors that affect an individual’s propensity to atherosclerosis, coronary thrombosis, electrical or autonomic instability can theoretically affect the risk for sudden death. Therefore, pathways that affect cholesterol metabolism, atherosclerotic plaque formation, the coagulation cascade, ion channels, gap junctions, receptor and signaling pathways, central and autonomic neural networks among many others can have a bearing on occurrence of sudden death. Allelic variations in an individual’s genetic make-up in these diverse yet interlinked physiological pathways can contribute to increased susceptibility to fatal ventricular arrhythmias^2^,^5^. The challenge is to identify the more common mutations/polymorphisms that increase susceptibility to ventricular arrhythmias.

The evidence that genes have a role to play in sudden death due to coronary artery disease has gathered from family studies. Studies on different ethnic populations have documented an increased predisposition to sudden death as a specific mode of death in certain families. In the Paris Prospective Study, there was a two-fold increase in the risk of sudden death if there was a parental history of sudden death^4^. This risk became nine times higher if both parents had a history of sudden death. Similar studies conducted in Seattle and in the Netherlands have documented an increased risk of sudden death due to fatal ventricular tachyarrhythmias in certain families^9^,^10^. This specific increase in the risk of sudden cardiac death is independent of the risk of myocardial infarction due to atherosclerosis. The effect of allelic interactions is evident from the wide variation in risk depending on parental history of sudden death. These allelic variations are the current subject of research and are elaborated upon in the next section.

## Genomics and sudden death

While classical Mendelian genetic disorders result from mutations in single genes that have relatively high penetrance and are therefore readily recognizable clinically, many common clinical disorders known to have hereditary transmission are associated with genetic mutations that do not produce a clinically recognizable phenotype due to poor penetrance yet are far more prevalent in the general population. Therefore, from a public health point of view, these mutations are more important than other rare mutations that may also result in an identical clinical picture. This is well illustrated from the contribution of various genetic mutations in the occurrence of Alzheimers disease^11^. While mutations in presenilin 1, presenilin 2 and β-amyloid precursor protein gene are highly penetrant causes of early-onset Alzheimer’s disease, these together account for less than 1 per cent of cases of Alzheimer’s disease in the general population. On the other hand, while polymorphims in the apolipoprotein E e4 allele are associated with a milder risk of late-onset Alzheimer’s disease, this variation contributes by far more cases in the general population than the other mutations due to its higher prevalence.

As the human genome has now been deciphered, it is possible today to identify potential genes that could influence expression of common multi-factorial diseases. It is well established that 99.9 per cent of genes between any two random individuals are similar. All the variation between individuals is based on a variation of 0.1 per cent in the gene pool, which accounts for approximately 3 million base pairs^12^. These variations, referred to as single nucleotide polymorphisms (SNP) account for majority of acquired diseases. Such SNPs and haplotypes, which represent a longer sequence of nucleotides, may directly be responsible for health and disease or may be a marker for a particular disease due to its proximity to the disease causing mutation. Systematic study of population based genetic information using linkage mapping, association studies or a candidate gene approach may help identify genetic variations associated with clinical disorders.

Variations in SNPs near numerous candidate genes that affect electrogenesis and propagation, autonomic tone, cardiac receptors and post-receptor signaling pathways along with atherothrombosis have been studied^13^–^18^. Alterations in these pathways could in some way predispose the patient to arterial thrombosis and ventricular tachyarrhythmias. For example, the S1102Y variant of the *SCN5A* gene predisposes individuals to arrhythmias in presence of QT prolonging drugs and hypokalemia^13^. While this variant is common in Africans it is not encountered in those of European and Asian descent. Other allelic variations have been studied that explore mutations in various other ion channels in different families^19^.

Effect of allelic variations affecting sympathetic tone on sudden death has been demonstrated in two studies. In both the Cardiovascular Health Study as well as the Cardiac Arrest Blood Study, the Q27E variant of the beta 2 receptor was associated with more sudden deaths than the other common variant, the G16R receptor^14^,^15^. The DD variant of the *ADRA2B* gene, which mediates coronary vasoconstriction, was associated with a higher risk of sudden death in the Helsinki Sudden Death Study^16^.

Polymorphisms in genes associated with thrombotic pathways have also been investigated. The 4G/5G polymorphism of the plasminogen activator inhibitor 1 gene has been associated with reduced fibrinolysis^17^. Similarly, the A2 polymorphism of the platelet glycoprotein IIIa receptor was associated with a higher risk of sudden death in those younger than 50 years in the Helsinki Sudden Death Study (Odds Ratio 2.5; 95% Confidence Interval 1.2–5.3)^18^.

Polymorphisms in many other biologic pathways that have a bearing on the pathogenesis of sudden death are being systematically evaluated in ongoing studies. To identify all possible genetic factors in a given population, ideally, large case-control studies involving thousands of cases and controls would be required to locate allelic variations associated with sudden death.

## Limitations

While genomics has opened up an exciting new dimension for assessing a patient at risk for sudden death, important limitations persist. Most of the current studies are of inadequate power due to small sample size. There are many false positive and false negative studies with issues of non-reproducibility of published data^19^,^20^. There is also the issue of publication bias with only positive studies being reported^19^,^20^. With significant variations being introduced by ethnicity and different heritability patterns in the young and the old, it is imperative to look into the study design before making overarching conclusions.

## Conclusions

Genes play a modest role in determining risk for common arrhythmias. Several genes and chromosomal loci have been identified for rare arrhythmias such as Brugada Syndrome, arrhythmogenic right ventricular dysplasia, long QT syndrome, *etc*. In addition, genomics will permit recognition of additional genetic mechanisms that affect the risk of sudden death in patients with coronary artery disease. These inherited variations will lead to a continuum of risk, varying with the allelic genotype. Greater understanding of genetics will also lead to more rational drug therapy. However, the role of gene therapy is still evolving and will need evaluation for long-term viability and effectiveness.
